# Chemical modification of AAV9 capsid with *N*-ethyl maleimide alters vector tissue tropism

**DOI:** 10.1038/s41598-023-35547-0

**Published:** 2023-05-25

**Authors:** Patrick L. Mulcrone, Anh K. Lam, Dylan Frabutt, Junping Zhang, Matthew Chrzanowski, Roland W. Herzog, Weidong Xiao

**Affiliations:** 1 Department of Pediatrics, Herman B Wells Center for Pediatric Research, Indiana University School of Medicine, Indianapolis, IN 46202 USA; 2 Lewis Katz School of Medicine, Temple University, Philadelphia, PA 19140 USA

**Keywords:** Biotechnology, Chemical biology, Molecular biology, Chemistry

## Abstract

Although more adeno-associated virus AAV-based drugs enter the clinic, vector tissue tropism remains an unresolved challenge that limits its full potential despite that the tissue tropism of naturally occurring AAV serotypes can be altered by genetic engineering capsid vie DNA shuffling, or molecular evolution. To further expand the tropism and thus potential applications of AAV vectors, we utilized an alternative approach that employs chemical modifications to covalently link small molecules to reactive exposed Lysine residues of AAV capsids. We demonstrated that AAV9 capsid modified with *N*-ethyl Maleimide (NEM) increased its tropism more towards murine bone marrow (osteoblast lineage**)** while decreased transduction of liver tissue compared to the unmodified capsid. In the bone marrow, AAV9-NEM transduced Cd31, Cd34, and Cd90 expressing cells at a higher percentage than unmodified AAV9. Moreover, AAV9-NEM localized strongly in vivo to cells lining the calcified trabecular bone and transduced primary murine osteoblasts in culture, while WT AAV9 transduced undifferentiated bone marrow stromal cells as well as osteoblasts. Our approach could provide a promising platform for expanding clinical AAV development to treat bone pathologies such as cancer and osteoporosis. Thus, chemical engineering the AAV capsid holds great potential for development of future generations of AAV vectors.

## Introduction

Recombinant adeno-associated virus (rAAV) gene therapy has a broad clinical impact on improving patient outcomes for various genetic diseases, including the three FDA-approved AAV-drugs: Luxturna (treatment for Leber congenital amaurosis), Zolgensma (treatment for spinal muscular atrophy), and recently Hemgenix (treatment for Hemophilia B)^1^. More than one hundred clinical trials using AAV drugs have been conducted to correct human defective genes, making AAV the most popular gene delivery system, mostly due to their relatively low immunogenicity, customizable design, and safety profile. Despite the fast pace of success for AAV vectors, unresolved challenges still exist such as limited tropism, poor transduction efficacy in certain tissues, pre-existing neutralizing antibodies (NAbs), and high vector dosage^2^.

Although design of the recombinant AAV vector promoter and transgene play an important role in determining which tissues and cell-types are targeted for transgene expression, vector tropism is fundamentally determined by the capsid used^3,4^. Wild-type (WT) AAV serotypes exhibit different tissue tropism due to different capsid protein sequences or variable regions interacting with disparate cell surface receptors, and thus can be utilized for different target tissues^3^. However, their tropism remains somewhat broad and unspecific, yet limited for certain tissues of interest^2^. In addition, pre-existing NAbs in the human population against certain AAV serotypes can compromise the patient’s transduction efficiency or eligibility for treatment. To overcome this, modifications to the AAV capsid have been investigated worldwide to expand the potential use for this virus. Many newly engineered capsids were developed from exploratory research by genetic modification of the cap gene to chemical approaches, yet more research is needed to understand the AAV capsids’ precise chemistry and their receptor binding interactions^4,5^. Several groups have made promising discoveries concerning capsid engineering via genetic engineering, directed and structure-guided evolution^6–8^, and ligand tagging or shielding the capsid through chemical modification^5,9,10^.

The viral capsid of AAVs is comprised of three viral proteins that share an overlapping reading frame: VP1, VP2, and VP3. Modified versions of AAVs range from genetic alterations of the viral cap gene to conjugation of proteins such as streptavidin or antibodies to promote interaction with specific cells. Previous work shows that adding distinct genetic sequences to the VP2 gene can alter the targeting of AAV9 to various cells within bone tissue and the transduction efficiency of AAV6 among a range of different cancer cells^11–14^. Indeed, Lee and Ahn showed that AAV2 modified with a streptavidin–biotin complex linked to an anti-EpCAM antibody was substantially effective at targeting EpCAM-positive ovarian cancer cells^15^. Given the successes documented by these capsid modifications, we hypothesized that altering the AAV capsid with small molecules would alter its surface receptor binding motif and therefore have an effect on the AAV tissue tropism and biodistribution.

Here, our study utilized a chemical modification approach to covalently link small molecules to reactive exposed Lysine residues of capsids AAV2, AAV8 and AAV9. From in vitro transduction screening, AAV9 capsid modified with *N*-ethyl Maleimide (NEM) was shown to best enhanced the gene expression of in human endothelial cell line, therefore NEM was chosen for further in vivo analyses in this study. Herein, we demonstrate that AAV9-NEM increased the transduction of bone marrow while decreased transduction of liver tissue in vivo, compared to unmodified WT AAV9, and that chemical engineering the AAV capsid with small molecules could bring great potential for future generation of AAV vectors.

## Results

### Development and characterization of AAV9-NEM

**Figure 1:**
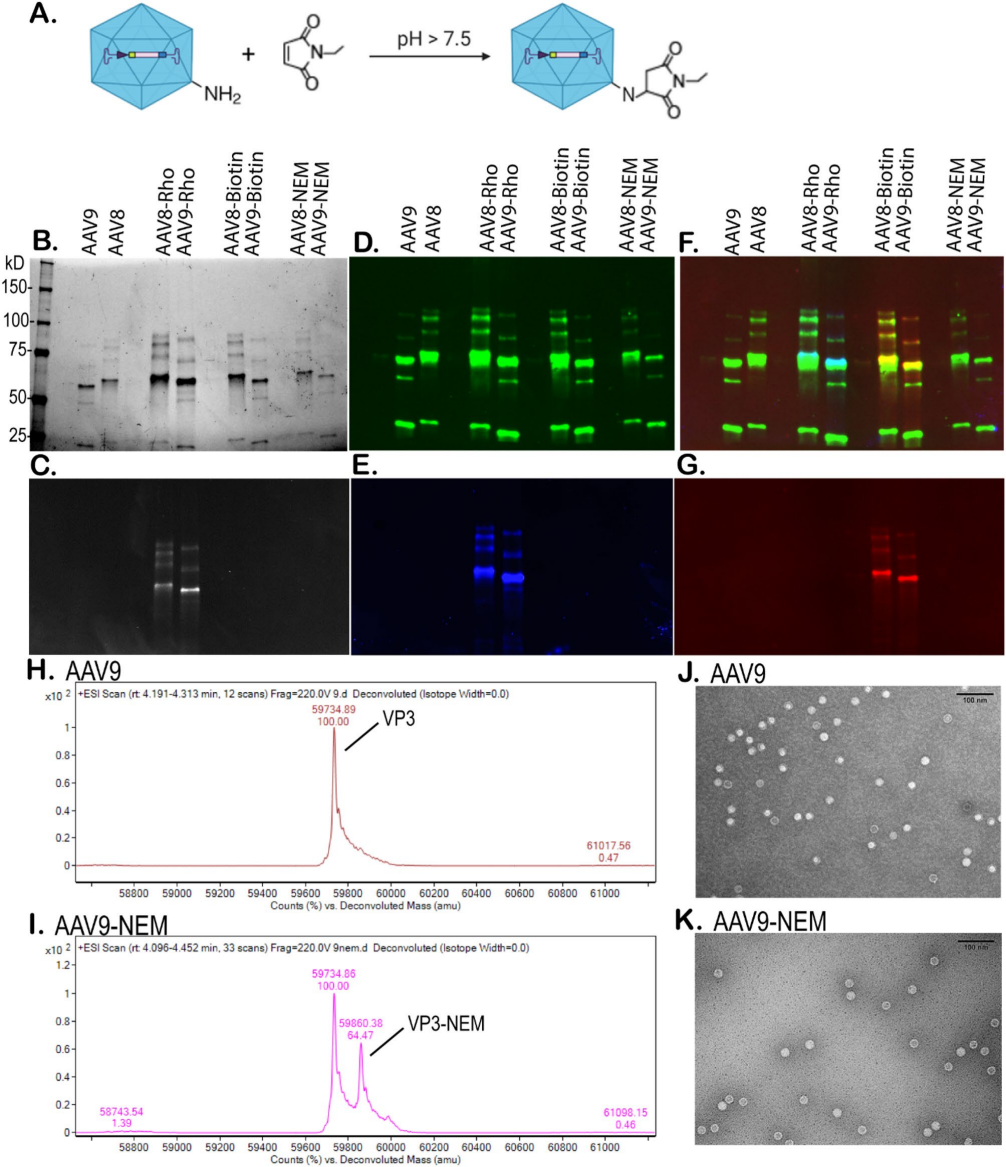
Characterization of chemically engineered capsid AAV9-NEM compared to its unmodified counterpart AAV9. Schematic diagram of AAV capsid 9 reacts with NEM molecules (**A**). SDS-PAGE of AAV8, AAV9, and different chemically modified capsids using 7.5% gel: total protein, silver staine of one gel is shown (**B**), and under rhodamine fluorescent excitation (**C**). Western blot of the same blot is shown: (**D–G**) with anti-VP1/2/3 (**D**), while the same blot under rhodamine fluorescent excitation (**E**), merged (**F**), or anti-biotin/streptavidin; Gel and blots (**B–G**) were imaged using *C*hemiDoc MP Imaging System (Bio-Rad) (**G**). Deconvoluted mass spectra (**H–I**) of AAV9 VP3 (**H**) and AAV9-NEM VP3 (**I**). Transmission electron micrographs (**J–K**) of AAV9 (**J**) and AAV9-NEM (**K**), scale bar = 100 nm.

Utilizing chemical modification strategies, AAV serotypes 8 and 9 were reacted with several different small molecules bearing the functional group of maleimide under basic condition (pH of 8). Any exposed lysine residues on the capsid of AAV8 and AAV9 would form a covalent bond with the maleimide molecules under this condition. Extensive dialysis was performed before the final modified AAVs were characterized. Figure 1A shows a schematic diagram of this reaction between the primary amino group of lysine and the *N*-ethyl maleimide (NEM). To confirm and characterize this reaction, different side chains of maleimide were tested including tetramethyl-rhodamine-5-maleimide (Rho) and biotin-maleimide (Biotin). As shown in Fig. 1B, a silver-stained SDS-PAGE gel showed the 3 distinct bands for VP1/2/3 proteins of AAV8, AAV9, and their modified capsids with VP3 being the most abundant. Figure 1C shows that this very same gel was previously imaged under the Ex/Em condition for Rhodamine provided only signal for the modified capsids of AAV8-Rho and AAV9-Rho. Figure 1D–G are Western blots confirming specific AAV VP1/2/3 proteins (Fig. 1D), direct Rhodamine fluorescent condition (Fig. 1E), or anti-biotin/streptavidin (Fig. 1G). Clearly, SDS-PAGE and Western blots indicated that the reaction of Rho- or Biotin-maleimide with the capsid AAV8 and AAV9 occurred, but these methods cannot confirm the reaction of the capsid with NEM (the smallest molecule tested with no indicating fluorescent marker). To do so, mass spectrometry with our optimized protocol^16^ was able to confirm this reaction. As shown in Fig. 1H, the deconvoluted mass spectrum of AAV9 VP3 shows the exact mass detected (59,734 Da), which almost exactly matches its theoretical mass (59,733 Da). Fig. 1I shows the same spectrum for AAV9-NEM VP3, and the exact mass gain of one NEM molecule was detected to be 59,860 Da, which confirmed the modification of NEM on AAV9. General morphology of AAV9-NEM compared to its unmodified counterpart AAV9 by transmission electron microscopy (TEM), revealed similar morphology among the two variants (Fig. 1J–K).

### In vitro evaluation of AAV9-NEM’s transduction

**Figure 2:**
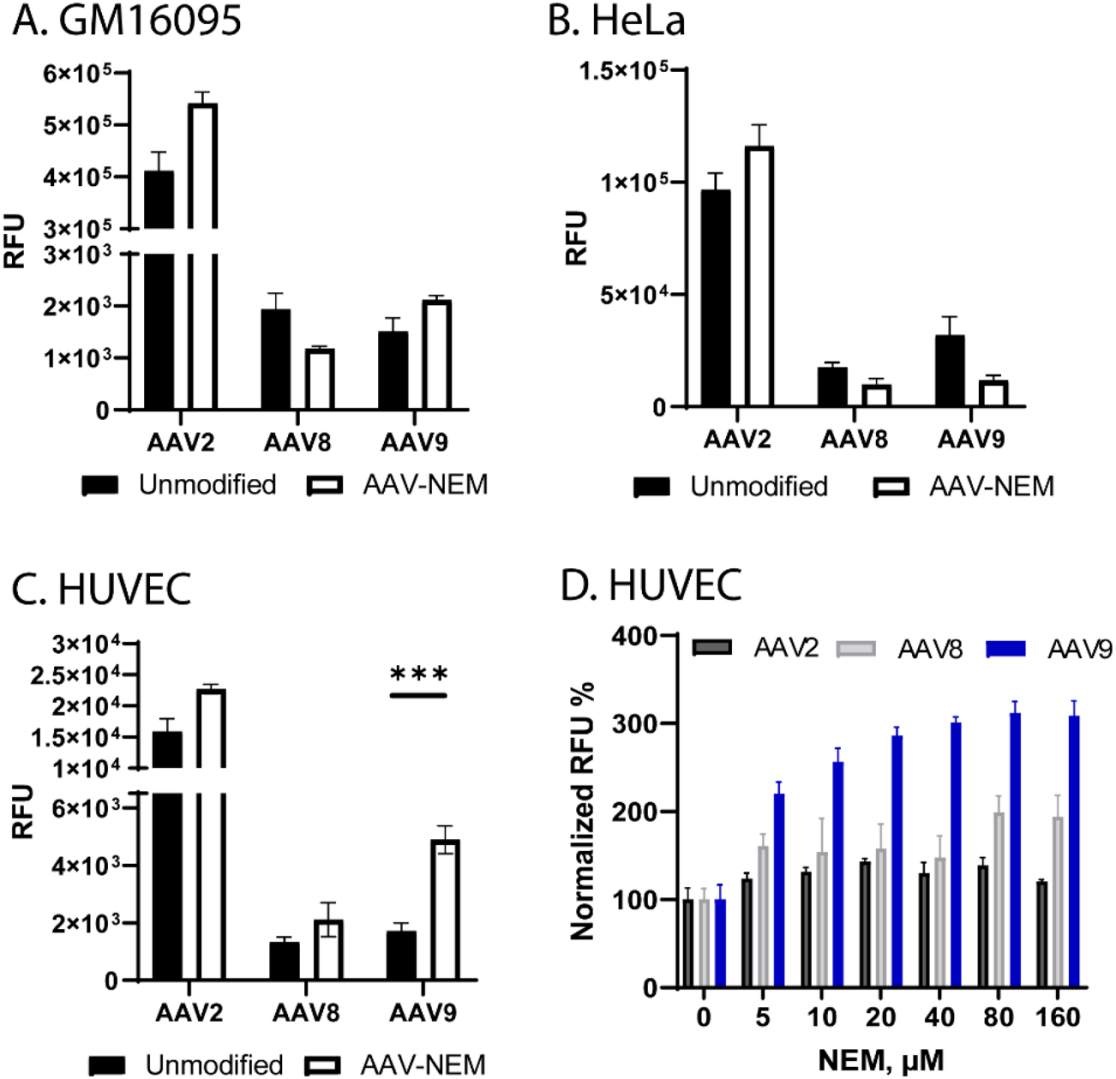
In vitro transduction assays of AAV WT vs NEM-modified capsids. gLUC signal was measured at 24 h-post transduction. (**A**) Human fibroblasts GM16095 cells. (**B**) Human cervical cancer HeLa cells. (**C, D**) Human umbilical vein endothelium HUVEC cells. (**D**) Dose-dependent increase in gLUC observed in HUVECs transduced with AAV9-NEM. N = 3/group.

To assess the phenotypic change of AAV-NEM, in vitro transduction assays were performed by infecting the same multiplicity of infection (MOIs) of AAV-NEM and their unmodified counterparts on different cell lines (GM16095, HeLa, and HUVEC). All of the vectors tested (AAV2, AAV8, and AAV9) packaged (pAAV-CB-gLUC) as indicated by the outcome measurements of Gaussia luciferase. As shown in Fig. 2A, B, no significant difference in transduction was measured on human fibroblast cells GM16095 and HeLa cells, respectively. However, the transduction of AAV9-NEM on human umbilical vein endothelial cells (HUVEC) was significantly higher than the WT AAV9 (Fig. 2C, D). Given these observed differences in vitro, along with no morphological differences between unmodified and NEM-modified AAV9 (Fig. 1J–K), we chose next to examine transduction patterns of our lead vector, AAV9-NEM, in vivo.

### In vivo evaluation of AAV9-NEM’s transduction and tropism

**Figure 3:**
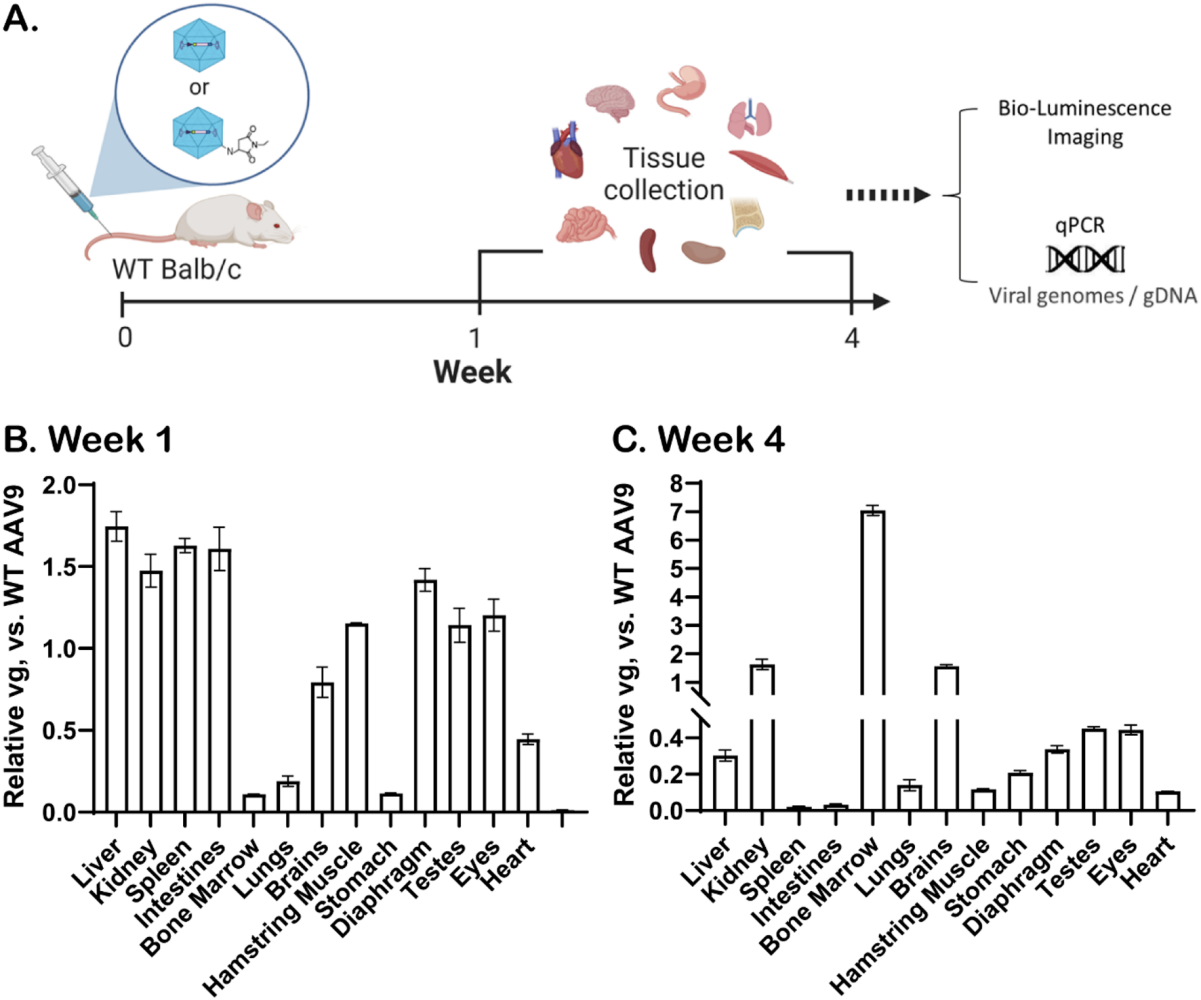
In vivo biodistribution of AAV9-NEM. In vivo study design of AAV9 vs AAV9-NEM systemic delivery via tail-vein injection of WT Balb/c mice (**A**). Biodistribution of AAV9-NEM at week-1 post injection (**B**). Biodistribution of AAV9-NEM at week-4 post injection (**C**). N = 2 mice/group.

Biodistribution analyses of AAV9-NEM compared to unmodified AAV9 were further evaluated by systemic delivery in WT Balb/c male mice. An illustration of the study design was shown in Fig. 3A, in which the same dose of AAV9 and AAV9-NEM (5 × 10^10^ vg per AAV; each capsid packaging a unique barcode for retrospective analysis) was injected IV. At week 1 or week 4 post injection, the mice were sacrificed for tissue collection and analyses.

As shown in Fig. 3B, DNA analysis of tissues at week 1 show the early changes in tropism of AAV9-NEM vs. AAV9, as that AAV9-NEM moderately increased vector DNA in some tissues (e.g., liver, kidney, or spleen) while decreased in others (e.g., bone marrow, lungs, or stomach). At week-4 post-injection, the change in tropism of AAV9-NEM was found to be more enhanced in the bone marrow, where vector genome (vg) DNA was around sevenfold higher than the unmodified AAV9 (Fig. 3C). The absolute vg detected corresponding to Fig. 3B is reported in Fig. S1. To examine the gene expression (gLUC), in vivo bioluminescence imaging was used with IP injection of the substrate coelenterazine with a dose of 200 mL/mouse (30 mg/mouse) and performed immediately after the substrate injection*.* AAV9-NEM gave a brighter signal (double the light intensity emitted) compared to AAV9 at week-4 post injection (Supplemental Fig. S2). These results indicate that the chemical-engineered capsid AAV9-NEM indeed changed the tropism and also enhanced the transduction of wild-type capsid AAV9.

### AAV9-NEM transduces separate populations of cells in murine livers and bones compared to WT AAV9

**Figure 4:**
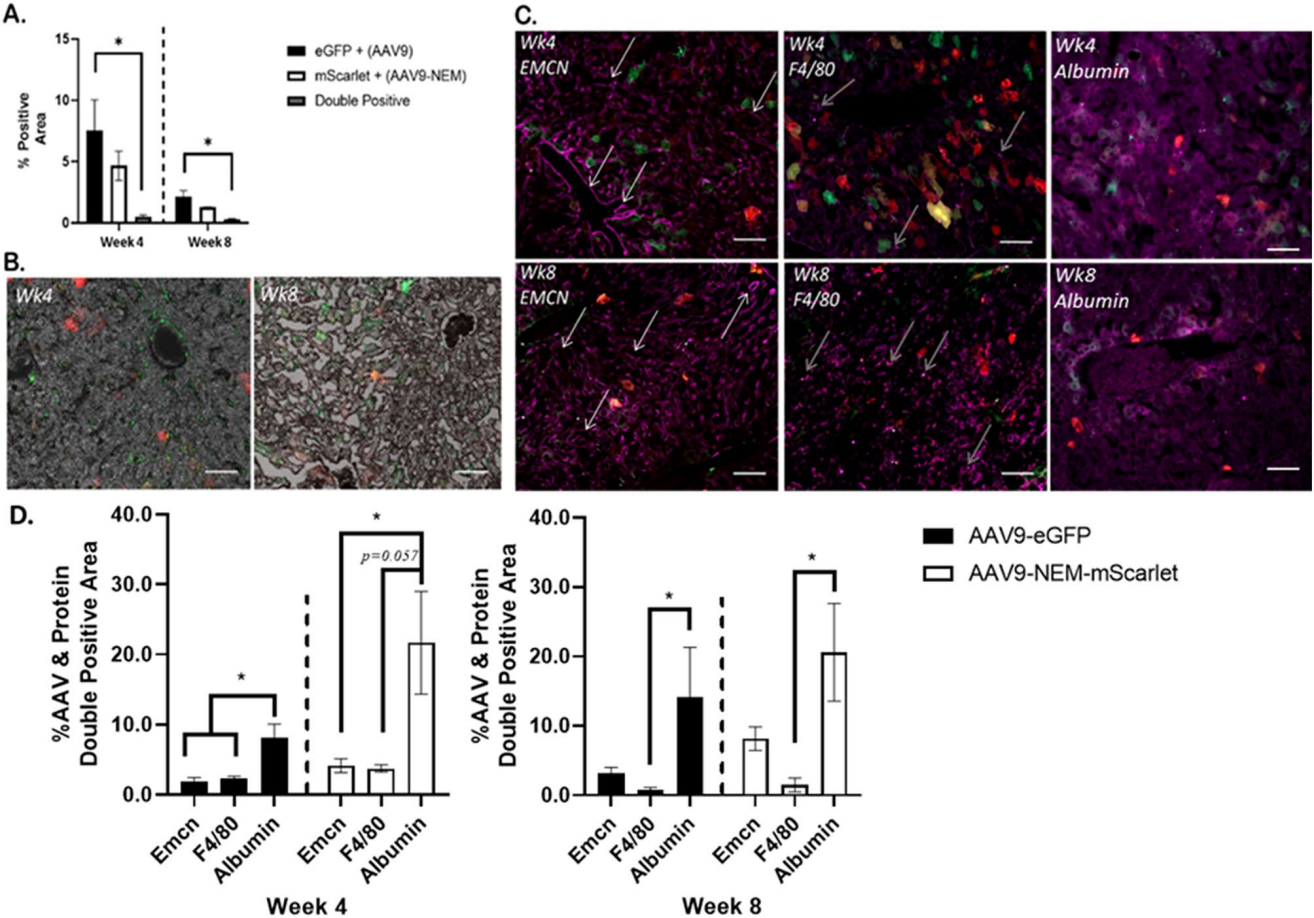
AAV9 (eGFP; Green) and AAV9-NEM (mScarlet; Red) transduce separate populations in murine liver. (**A**) eGFP and mScarlet positive area of mouse livers. Double positive areas were minimal at 4- and 8-week timepoints. (**B**) Representative 10× images of (**A**) quantification. eGFP = AAV9, mScarlet = AAV9-NEM. (**C**) Immunofluorescent staining for Endomucin (Emcn), F4/80, or Albumin (Purple) along with AAV9 (eGFP) and AAV9-NEM (mScarlet) cells in serial liver sections. White arrows indicate sinusoidal, Emcn-positive vascular structures, and gray arrows identify some puncta (Kupffer Cells) that are F4/80 positive. Scale bars = 50 µm. (**D**) Quantification of AAV9 (eGFP) or AAV9-NEM (mScarlet) positive liver tissue that is Emcn, F4/80, or Albumin positive. Comparisons via One-way ANOVA, but Week 8 AAV9-eGFP data compared by Kruskal–Wallace test. AAV dose for the mice is 1.89 × 10^11^ vg/kg.

To further investigate observed differences between AAV9 and AAV9-NEM detected in the livers and bone marrow, we designed experiments using fluorescent proteins to compare transduction of specific regions of these two organs. We chose AAV9-eGFP (packaged pdsAAV-CB-eGFP) and AAV9-mScarlet (packaged pdsAAV-CB-mScarlet) modified with NEM for the in vivo study, as well as lengthened the time course to better understand the dynamics of NEM modification of AAV9^17^. At 4- and 8-weeks post injection, soft organs and hindlimbs were collected and processed for histological, fluorescent, and flow cytometry analyses. Liver sections revealed a slight decrease in AAV9-NEM positive cells, indicated by mScarlet expression, compared to AAV9-eGFP transduced cells (Fig. 4). This trend matches the vg content data presented in Fig. 3. At 8 weeks, both eGFP and mScarlet positive cell numbers decreased, yet at both timepoints, there were only few double positive eGFP and mScarlet liver cells. When liver sections were stained for the vascular marker Endomucin (Emcn) and the Kupffer cell/macrophage marker F4/80, there was minimal overlay between these antibodies and both populations of AAV9-transduced cells compared to when sections were stained with hepatocyte marker Albumin; this was observed at 4 weeks and 8 weeks post injection^18,19^. This data suggests that most of the cells being transduced with AAV9 in the liver are most likely hepatocytes and not endothelium or phagocytic cells present in the tissue^20–22^.

**Figure 5:**
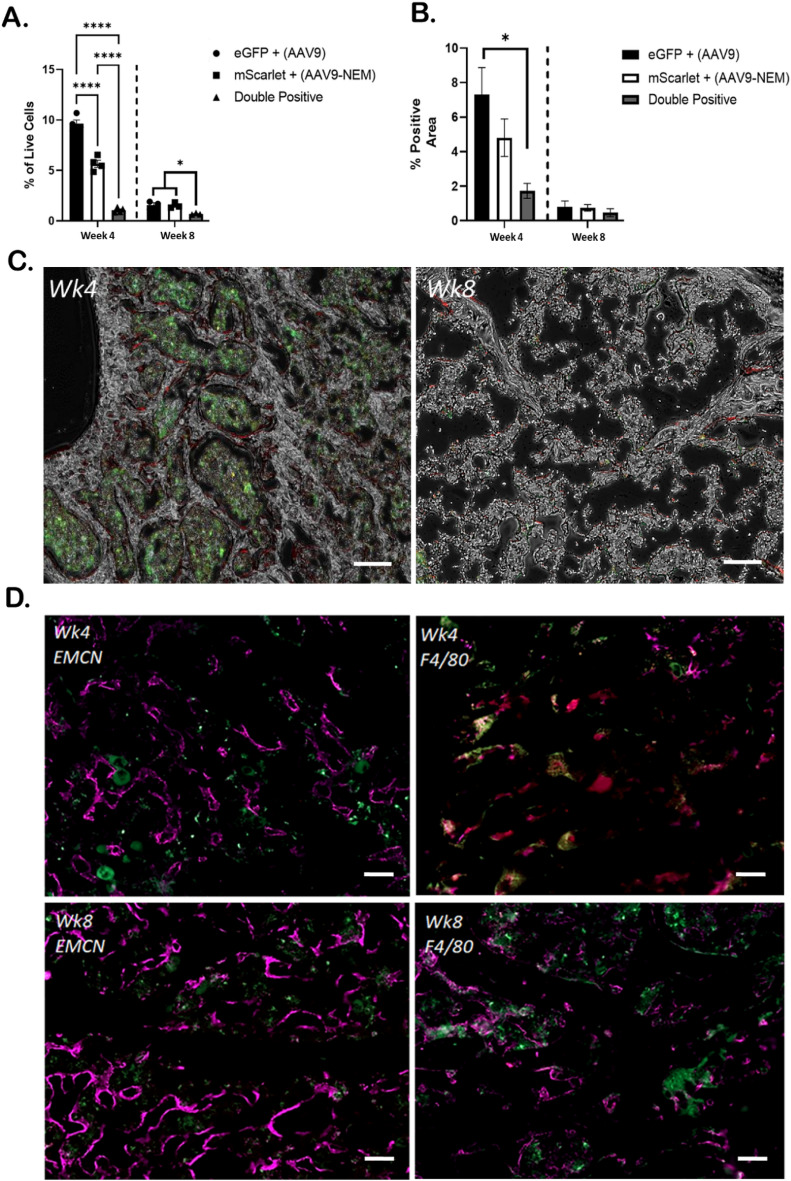
AAV9 and AAV9-NEM transduce separate populations in murine bone marrow. (**A**) Flow cytometry analysis of live eGFP, mScarlet, and double positive bone marrow cells. (**B**) GFP and mScarlet positive area of mouse femurs. Double positive areas were minimal at 4- and 8-week timepoints. (**C**) Representative 10× images. Notice the localization of eGFP (marrow) and mScarlet (bone lining interface) eGFP = AAV9, mScarlet = AAV9-NEM; scale bars = 100 µm. (**D**) Immunofluorescent staining for Endomucin (Emcn) or F4/80 (Purple) along with AAV9 (eGFP) and AAV9-NEM (mScarlet) cells; scale bars = 50 µm. One-way ANOVA for comparisons. N = 4 for 4-week experiment, N = 3 for 8-week experiment. AAV dose for the mice is 1.89 × 10^11^ vg/kg.

In the bone marrow, we observed a 4% and a 2.5% difference between AAV9-eGFP and AAV9-NEM-mScarlet via flow cytometry and immunofluorescence, respectively, at 4 weeks post injection (Fig. 5A, B). As we observed in the liver, the number of double positive cells in the bone marrow was significantly lower compared to the single-positive groups. We observed a similar difference in bone marrow at 4 weeks when the fluorescent transgenes were exchanged between the AAV9 vectors (Supplemental Fig. S3). By 8 weeks, all populations in the bone marrow decreased to below 2% using both methods of analysis. Of note, fluorescent imaging showed distinct areas of green and red positivity in the hindlimbs; the marrow component contained strong eGFP green signal, and a prominent mScarlet red signal was observed at the interface between the calcified tissue and the marrow tissue, suggesting distinct populations of bone cells are being transduced with the AAV9-NEM vs. AAV9 (Fig. 5C). Indeed, flow cytometric analysis shows that AAV9-NEM-mScarlet cells exhibited a higher % of single-positive cells with vascular markers Cd31 and Cd34, and markers of MSCs with osteogenic potential Cd90, Cd105, and Cxcr4 at 4 weeks compared to AAV9-eGFP cells^23^; Cd31 and Cd90 differences persisted at the 8-week timepoint (Supplemental Fig. S4).

Given that Cd31, Cd34, and Cd90 are markers for vascular cells and to certain extent immune cells, we stained the bone sections with Emcn and F4/80 to investigate more detail about which cell types are being transduced^24,25^. Interestingly, Emcn overlayed strongly with mScarlet and less so with eGFP in our analysis at both 4 and 8 weeks. This pattern was not observed with F4/80; there was minimal co-localization with either eGFP or mScarlet populations (Fig. 5D). Overall, these results suggest that AAV9 transduction pattern is different between the liver and the bone marrow, and that the NEM modification further alters that pattern of AAV9 transduction.

### AAV9-NEM exhibits enhanced transduction of BMSCs pushed toward an osteoblast lineage

**Figure 6:**
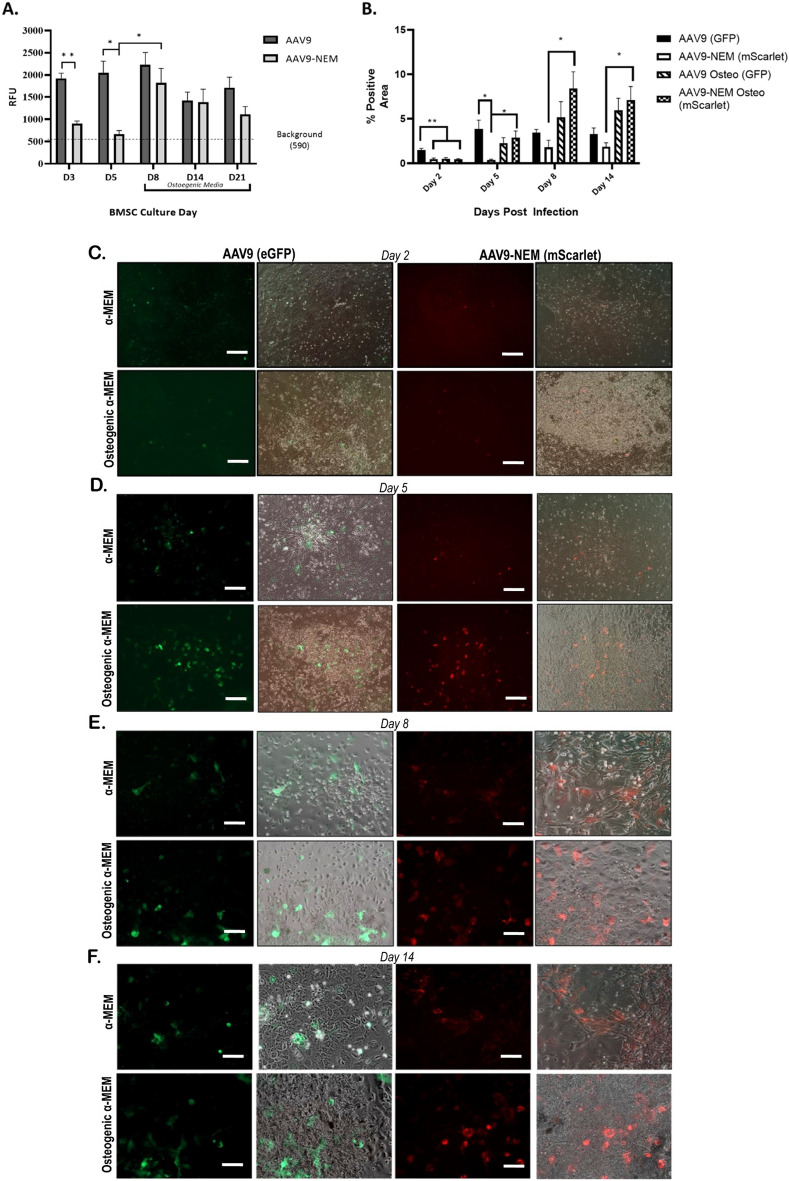
NEM modification Alters the Transduction of AAV9 in BMSCs differentiated to osteoblasts in vitro*.* (**A**) gLUC readout of primary murine BMSCs and osteoblasts 48 h post transduction. MOI of 10^4^ is the dose. N = 3–5 (**B**) % of red or green BMSCs over osteoblast differentiation experiment. AAV9-NEM-mScarlet area significantly greater starting at Day5 post injection, MOI of 10^5^. (**C–F**) Representative images of GFP and mScarlet cultures at Day 2 (**C**), Day 5 (**D**), Day 8 (**E**), and Day 14 (**F**). Scale bars = 100 µm in (**C, D**). Scale bars = 50 µm in (**E, F**). All analyses via One-Way ANOVA. N = 3, *p < 0.05, **p < 0.01.

The location of the mScarlet-positive AAV9-NEM cells in the bone tissues of the mice, and the presence of Cd90 and Cxcr4-positive AAV9-NEM bone marrow suggest that cells involved in bone turnover could be a target of this modified AAV9 vector^26^. To investigate these potential transduction differences between AAV9 and AAV9-NEM in a more controlled environment, we isolated primary mouse bone marrow stromal cells (BMSCs) from bone marrow of uninjected Balb/C male adult mice for two sets of studies. We employed AAV9 or AAV9-NEM driving expression of gLUC under control of the CB promoter for the first set of experiments, and AAV9-CB-eGFP along with AAV9-NEM-CB-mScarlet for the second set. At Days 3 and 5 of the gLUC experiments, BMSCs transduced with AAV9 (MOI of 10^4^) exhibited significantly higher luciferase readout compared to AAV9-NEM. Interestingly, BMSCs transduced on Day 8 (48 h in osteogenic medium) with either AAV9 or AAV9-NEM resulted in similar luciferase readings. This trend continued to be seen on Days 14 and 21 (Fig. 6A). This data suggests that the NEM modification of AAV9 alters its transduction in vitro*.*

For the second set of experiments, our goal was to elucidate more details about this observed difference in AAV9-NEM transduction in undifferentiated BMSCs compared to those pushed towards an osteoblast lineage. BMSCs were grouped based on AAV9 or AAV9-NEM transduction as well as growth in osteogenic medium or regular α-MEM medium after 7 days of initial growth in α-MEM. As we observed in the gLUC studies, AAV9-eGFP transduced the greatest percentage of BMSCs in α-MEM at Days 2 and 5 compared to AAV9-NEM-mScarlet in α-MEM or either of the osteogenic culture systems. On Day 5, however, the AAV9-NEM-mScarlet signal in the osteogenic culture was significantly greater than that seen in the AAV9-NEM-mScarlet α-MEM culture. This significant difference in red cells persisted on Days 8 and 14 of this experiment (Fig. 6B–F). In addition, the percentage of cells transduced with AAV9-eGFP steadily increased in osteogenic cultures, matching that of the α-MEM cultures; this observation is similar to our data in the gLUC study. Upon further analysis of both osteogenic cultures, a majority of transduced cells localized to the calcified nodules at Days 8 and 14 (Supplemental Fig. S5). Overall, the results from these studies suggest that the NEM modification on AAV9 can alter its transduction tropism in vitro in BMSC cultures, and that AAV9-NEM transduces osteoblastic cells more successfully than undifferentiated BMSCs in culture settings.

### AAV9-NEM-mScarlet and osteocalcin positive cells observed at the marrow/calcified bone interface

**Figure 7:**
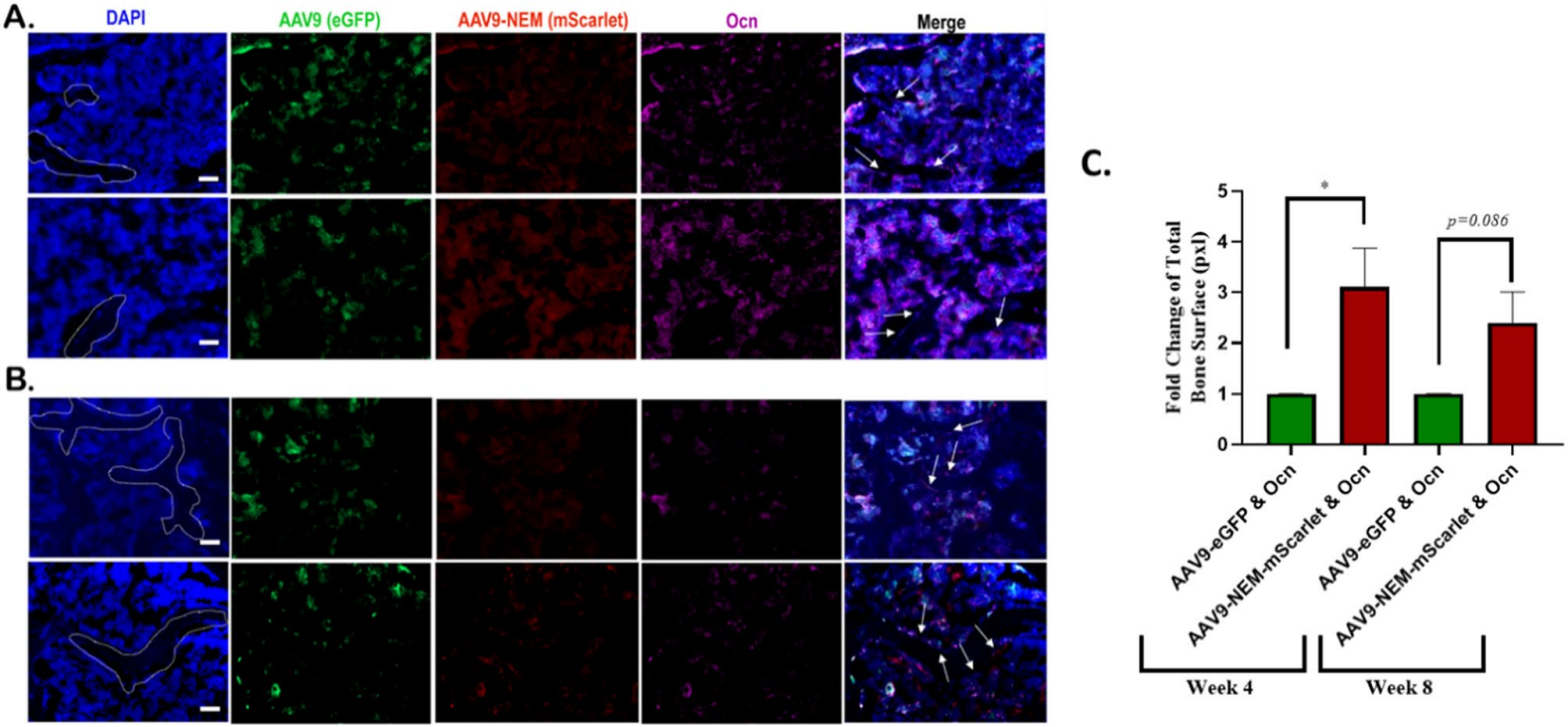
Osteocalcin-stained cells co-localize with AAV9-NEM-mScarlet more than AAV9-eGFP at the Marrow/Calcified Bone Interface. (**A**) 4-week bone marrow images. (**B**) 8-week bone marrow images. White dotted areas outline calcified bone tissue in DAPI stain. Osteocalcin (Ocn) stained with a Cy5-conjugated secondary antibody. White arrows on Merge indicate Ocn & mScarlet + osteoblasts. Scale bars = 50 µm. (**C**) Fold change of Ocn & mScarlet positive bone surface relative to Ocn & eGFP positive bone surface at 4 weeks and 8 weeks. N = 3–4/group, Ratio paired T-Test used. AAV dose for the mice is 1.89 × 10^11^ vg/kg.

Based on these in vitro data and the location of the mScarlet positive cells (Fig. 5B) in the bone sections, we chose to further analyze the in vivo study to assess co-localization of the osteoblast marker, osteocalcin (Ocn), with AAV9-eGFP and AAV9-NEM-mScarlet transduced cells in the liver and bone. Ocn, a secreted factor produced by mature osteoblasts that has various endocrine effects both in the bone and in other organs, was detected throughout the bone, in both the marrow as well as at the interface lining the calcified tissue at 4 weeks (Fig. 7A) and 8 weeks (Fig. 7B) post injection; no osteocalcin was observed in the livers (data not shown)^27,28^. Interestingly, co-localization between mScarlet-positive cells along the edge of the calcified bone and Ocn (purple) was consistently observed in the bone sections, suggestive of osteoblasts. Regarding eGFP positive areas, some Ocn co-localization is seen in the marrow area, but minimal eGFP/Ocn co-localization was observed along the edge of the calcified bone (Fig. 7C).

## Discussion

AAV gene therapy is proving to be an effective therapeutic option for various disease clinically. This vector can be designed to specifically target tissues or cells to correct genetic defects driving disease pathology. In addition to biological components of the AAV itself, modifications to the genome and the capsid proteins are expanding the application of AAV in basic research as well as pharmaceutical drug development. Here, we demonstrate that chemical modification of exposed lysine residues of the AAV9 capsid with *N*-ethylmaleimide (NEM) alters the transduction pattern and efficiency of AAV9 in vitro and in vivo.

To characterize this covalent modification of the AAV capsids, mass spectrometry was used to directly confirmed the mass gain of NEM molecule (Fig. 1). Despite having eight Lysine residues on the surface of each AAV9 VP3, the mass spectrum of AAV9-NEM indicated that in a majority of the reactions, only one Lysine per VP reacted to NEM molecules. Additionally, more than half of the capsid proteins are shown to still remain their original mass (Fig. 1I) which is indicative that they did not react to the NEM molecules; there might be possibilities that those reacted with NEM are from different lysine sites of the AAV9 capsid. This result shows that the AAV capsid is much more inert than we anticipated. Though more mass spectrometry is required to address the sites of reaction, the data here highlighted the power of mass spectrometry in confirming the capsid protein modification, since engineered capsids from previous studies were only indirectly characterized by Western blots using a different sidechain of the same functional group (fluorophore or secondary tags) to support the assumption that chemical reactions had happened^5,9,29,30^. The chemically engineered AAV9-NEM transduced HUVECs and murine BMSCs differentiated toward osteoblasts greater than WT AAV9 in culture. In the osteoblast cultures, a majority of the transduced cells localized to calcified nodules, indicative of osteoblastic cells. In a murine in vivo biodistribution study using a dose of 5E^10^, vector genomes delivered by AAV9-NEM were detected at higher concentrations in bone marrow and at lower concentrations in the liver 4 weeks post injection compared to WT AAV9 (Fig. 3). This result is indicative of the change in tropism by AAV9-NEM as the viral genomes delivered to the bone marrow were ~ sevenfold higher than the unmodified counterpart AAV9. In mice injected with the dual vector concoction (both the NEM-modified and WT vectors), AAV9-NEM and AAV9 transduced separate populations of cells in both liver and bone marrow as each single positive population in both organs was significantly greater than double-positive cells up to 8 weeks post injection (Figs. 4, 5). Interestingly, overall gene expression in the bone marrow by AAV9-NEM was not much enhanced (Fig. 5), which we did not expect based on the results of DNA analysis (Fig. 3). Even though the viral genomes were greatly found in the bone marrow, mechanisms involving vector uncoating or trafficking of AAV9-NEM may be different compared to AAV9 and will require more investigation. In bone marrow isolates from mice, a greater percentage of AAV9-NEM transduced cells compared to AAV9 transduced cells expressed Cd31, Cd34, or Cd90 (Supplemental Fig. S4). We detected elevated co-localization of Albumin-positive hepatocytes compared to minimal co-localization of F4/80+ Kupffer cells/macrophages or Emcn + endothelial cells with AAV-transduced liver tissue, while in the bone marrow, AAV9-NEM exhibited co-localization with Emcn and osteocalcin (Ocn) positive populations (Figs. 5D, 7). Many of the Ocn/AAV9-NEM double positive cells were located at the interface between marrow and calcified bone tissue (Fig. 7). Moreover, NEM modification of AAV9 altered in vitro transduction of primary BMSCs and those differentiated to osteoblasts (Fig. 6). Our results support the concept of AAV chemical modification as a way to alter tropism of AAVs and to expand their potential clinical and pathological applications.

There are many ways that a chemical modification to AAV could alter its biology, as multiple cell surface proteins and co-receptors interact with AAVs during trafficking and cellular uptake^31^. It is documented that various cell receptors and co-receptors are key for the AAV entry, and these receptors differ based on AAV serotype^32^. Intracellular trafficking of AAVs relies on the ubiquitin–proteasome pathway recognized by exposed lysine residues. From a previous study, Li et al*.* mutated surface-exposed lysines of AAV2 and AAV8 using site-directed mutagenesis, and only some lysine-mutated AAV2s gave significant increase in transduction efficiency of murine hepatocytes while mutated AAV8 did not^33^. Although we used a different approach, what we found in this study corroborates their results in terms of targeting surface exposed lysines to alter transduction and tropism pattern and that the modification is serotype-specific. For AAV9, galactose and Laminin Receptor 1 (RPSA) are known receptors for AAV9 and present in both liver and bone marrow^34^. Organs in mouse models that AAV9 transduces range from liver, pancreas, heart, and skeletal muscle to the hypoxic bone and the central nervous system protected by the difficult to traverse blood–brain barrier. Moreover, a cell surface receptor deemed AAVR is documented to interact with several serotypes, including AAV2 and AAV9^35^. With the addition of NEM to AAV9, affinity to these receptors may be altered, and the NEM may induce AAV9’s interaction with new receptors that are not accessible to WT AAV9. Indeed, researchers have made capsid modifications to avoid the immune system or to increase homing to tissues of interest^36^. As we chose to focus on endothelium, macrophages, and osteoblastic cells for the analysis of the AAV9-NEM, more comprehensive analyses of subsets of liver and bone cells in our study, especially in primary cells after in vivo transduction, will help elucidate AAV9-NEM transduced cells and provide clues about which receptors may be of future interest.

While our results using the reporter proteins gLUC, eGFP, and mScarlet indicate an altered transduction of AAV9-NEM in specific tissues, future studies with this viral vector need to explore this result in relevant disease models in which AAV9 has shown promise: spinal muscular atrophy, osteoporosis, and various neurological diseases^11,12,37,38^. Determining functional, clinically relevant enhancement of NEM-modified AAV using in vivo models at varying doses that span the range of safe vg injections would complement our present results and potentially lead to mechanisms of why the NEM modification indeed is changing AAV9 transduction and cellular uptake profiles^3,11–13,18,39^. Moreover, research into how the NEM modification alters the immune system reaction to AAV9 could elucidate possible mechanisms of the increased transduction observed in vivo. A key clinical issue regarding AAV gene therapy is the development of antibodies specific for the viral capsid and re-administration of the AAV in patients^40–42^. The development of these antibodies results in immune suppression of the AAV and decreased efficacy of the therapy. While immune targeting drugs are showing some promise in mitigating AAV-centric immune responses^39,43^, chemical engineering of AAVs could be an alternative. Recently, Yuan et al. demonstrate that chemically attaching phosphoserine moieties with a zwitterionic peptide to AAV8-FVIII reduced capsid-specific IgGs and T and B-cell activation; this also had no effect on FVIII-correction of Hemophilia A phenotype^44^, which emphasizes the immense potential of chemical engineering in AAV research.

Different from site-mutagenesis, one big advantage of chemical modification is that the AAV capsid was already packaged and purified before a chemical reaction takes place, which does not affect the packaging capability of the cap gene. This opens up endless opportunities for reacting the AAV capsids with small molecules, fluorophores, ligands, and even oligos. By combining with high-throughput phenotypic screening of in vitro or in vivo strategies, chemically engineered capsids can be filtered and chosen per purpose of interest. In conclusion, we report that a chemically engineered AAV9-NEM altered the transduction profiles of murine liver and bone tissues. Although more research is needed to clearly understand the mechanism of action behind these differences, our data suggests that chemical modifications of AAVs that maintain capsid integrity could be tailored to expand the biological applicability of this viral-based gene therapy.

## Materials and methods

### AAV construct design and rAAV production

The parent plasmid pDS-CB-EGFP has been described previously^18^. pDS-CB-mScarlet was synthesized by enzymatic removal of eGFP sequence from our in-house vector and the subsequent ligation of mScarlet sequence. The mScarlet transgene was ordered from IDT as a gbloc using the sequence from Bindels et al.^17^ and amplified using the PCR primers *eGFP.mscar.S* (gcggccgatccaccggtcgccaccaccatggtgagcaagg) and *bgh.mscar.A* (ataagcttgatggccgctttacttgtacagctcgtccatgccg). This PCR product was inserted into the NcoI/BsrGI digested pdsAAV-CB-eGFP vector using NEB HIFI assembly to produce pdsAAV-CB-mScarlet.

A triple plasmid co-transfection method was used to produce rAAVs as described previously^16,18^. One part vector plasmid, one-part specific AAV helper plasmid, and one-part mini adenovirus function helper plasmid, pFΔ6, were co-transfected into HEK293 cells cultured in roller bottles at a molar mass ratio of 1:1:1. The transfected cells were harvested 4 days later.

### AAV purification and titer determination

rAAV vectors were purified using liquid chromatography, followed a detailed protocol by Lam et al.^16^.

AAV vector genome titers were determined by qPCR/ddPCR assay following the previous protocol^18^. Briefly, 10 µLs of purified virus was treated in 90 µLs DNase I buffer (DNase I, 1U) at 37 °C for 1 h, then heated at 85 °C for 20 min to inactivate DNase I. Next, 50 µLs lysis buffer (direct qPCR lysis buffer) containing 0.5 mg/mL proteinase K was added, incubated for 1 h at 56 ℃ and heated at 95 °C for 15 min. rAAV genomes were amplified using various primers, provided in Table 1, and the titer for each was calculated. SnapGene software was used to document all vector processes, production of new vectors, and storage of plasmid maps and enzyme information.

**Table 1:** AAV titer primer and probe sequences.

Vector component	5ʹ Primer	3ʹ Primer	Probe
CB Promoter	cccatagtaacgccaatagggact	cccataaggtcatgtactgggcataa	ccacttggcagtacatcaagtgtatcatatgcca
eGFP	acatgaagcagcacgacttct	ttcagctcgatgcggttcac	ctacaagacccgcgccgag
mScarlet	ctgaagggcgacattaagat	ttgtgggaggtgatgtccaa	aagcccgtgcagatgcccggc

### Chemical modification of rAAVs

After optimizing the reactions, purified rAAVs (AAV8 and AAV9) were reacted with NEM, Biotin-maleimide, or Rhodamine-maleimide (Sigma) in PBS buffer at pH 8, with a molar ratio of 10,000 molecules/vg, overnight at 4 °C. The reacted samples were then filtered, and buffer exchanged extensively (~ 10 times) with PBS buffer pH 7.2 using Vivaspin20 100 K MWCO and concentrated down to around the initial volume of the rAAV. The chemically modified rAAVs were then titrated alongside with the un-modified rAAVs counterparts for titer determination before any downstream analyses and animal injections.

### AAV capsid characterization by LC–MS

LC–MS characterization of engineered capsids was followed precisely as detailed in our recent publication by Lam et al.^16^.

### SDS-PAGE and silver staining

Mini-PROTEAN TGX pre-cast gel (7.5% a polyacrylamide gel, Bio-Rad) was used. A sample of 6 µL rAAV (~ 1E12 vg/mL) was added to 2 µL of 4× Laemmli sample buffer containing 10% of β-mercaptoethanol, and mixed well before being heated at 90 °C for 5 min. The sample was cooled to room temperature and loaded into the gel lanes, together with a standard marker. Running buffer was 1× Tris/Glycine/SDS, Bio-Rad). The assembly was set and connected. Voltage was set to be constant, at 200 V. The gel was set to electrophorese for 30 min. The gel was removed from the cassette and stained using Pierce Silver Staining Kit, followed the manufacture’s protocol. The gel was imaged using ChemiDoc MP Imaging System (Bio-Rad).

### Western blot

The gel from SDS-PAGE was removed and transferred onto a nitrocellulose membrane using the Trans-Blot Semi-Dry Transfer Cell, Bio-Rad. Transfer buffer was 1× Tris/Glycine/SDS, 20% methanol. The cell was run at 0.16A (constant A) for 30 min. The blot was then blocked with Intercept Blocking Buffer, LI-COR, on a rocking platform for 30 min, and washed 3× with PBS (0.01% Tween 20); each wash took 5 min on a rocking platform. The blot was incubated with primary antibody LICOR-IRdye-680RD Streptavidin (1:5000 dilution) for 1 h at room temperature and then with Anti-AAV VP1/2/3 mouse IgG1-B1 #03-65158 (1:500 dilution) overnight at 4 °C on a rocking platform, and washed 3× with PBS (0.01% Tween 20). Secondary antibody incubation was LICOR-IRdye800 donkey anti-mouse IgG (1:20,000 dilution) for 1 h at room temperature, and washed 3× with PBS before being imaged on ChemiDoc MP Imaging System (Bio-Rad).

### In vivo mouse studies with rAAV9

All animals were maintained in the laboratory animal resource center at Indiana University–Purdue University, Indianapolis (IUPUI). All animal experimental protocols were approved and performed as per the guidelines of Indiana University’s Institutional Biosafety Committees (IBC) and Institutional Animal Care and Use Committee (IACUC) and in accordance with ARRIVE guidelines. Each specific experiment (specified in each Results Figure) was designed so that each vector (AAV9 or AAV9-NEM) packaged a unique transgene of interest so that they allowed for different purpose of analyses. For the Biodistribution study, AAV9 and AAV9-NEM each carried a unique barcode for retrospective analysis of the viral genome which determined the capsid tropism. DNA extractions from mouse tissues were performed using the Qiagen DNeasy Blood and Tissue Kit per manufacturer’s protocol. For the AAV9-eGFP/ AAV9-NEM-mScarlet in vivo study, 8–10 week old male Balb/C mice were intravenously injected via the tail vein with 5 × 10^10^ vg (viral genomes) with rAAV9/rAAV9-NEM concoction. This dose approximately equates to 1.89 × 10^11^ vg/kg based on the average weight of 26.5 g for the mice at injection used in these studies. PBS was injected into Mock animals. Prior to injection, mice were weight and placed under a heat lamp for 10 min to dilate vessels to ease injections and then monitored for 30 min post injection for any complications. At sacrifice, we measured a final body weight as well as liver and spleen weights before euthanasia (using isoflurane via inhalation) and soft tissue collection with no abnormal observation (Supplemental Fig. S6).

### BMSC differentiation

Primary bone marrow stromal cells from 8 to 10-week-old male Balb/C mice was performed as previously described^19,45^. Briefly, mouse hindlimbs were extracted, stripped of fascia and tissue, and the proximal epiphyses were cut off. Bones were inserted into 0.5 mL tube punctured at the bottom that was placed into 1.5 mL tube containing sterile 1× PBS. Bones were centrifuged, and the resulting pellets were grown in 10% α-MEM in 24 well plates. For wells selected for osteogenic differentiation, osteogenic medium (10% α-MEM containing 50 µg/mL ascorbic acid [Sigma, #A-5950] and 5 mM beta-glycerophosphate [Sigma, #G9891-25G]), was used for up to 20 days.

### In vitro cell line experiments

For Luciferase in vitro experiments, rAAV9 or rAAV9 modified with NEM driving expression of Guassia Luciferase (gLUC) was transduced into cells at MOI of 10^4^. For experiments with BMSCs, luciferase readout was conducted 48 h post transduction on days 3, 5, 8, 14, and 21 of culture. Osteogenic media was started on day 6. Cell media was collected, and 20 μLs were placed into wells of an all-white, 96-well plate. 50 μLs of 1× coelenterazine was added using a multichannel pipet, and plate was read immediately for luminescence after a brief shake at an integration time of 10 ms.

For eGFP and mScarlet in vitro experiments, primary BMSCs were transduced with rAAV9-eGFP or rAAV9-NEM-mScarlet at MOI of 10^5^ after reaching confluency at D14 of culture. After AAV transduction, certain BMSCs were cultured in osteogenic media for the remainder of the study. Fluorescent images were taken at Days 2, 5, 8, and 14 post-transduction. 3–4 images per group were analyzed for % green or red positive area via Metamorph Software. Calcified nodules were noticeable 48 h after starting the osteogenic media cultures.

GM16095 human fibroblasts (Coriell Institute), HeLa cells (ATCC), and HEK293s (ATCC) were all grown in 10% DMEM with 1% antibiotics. HUVEC were kindly provided by the Corson Lab (IUSM) and cultured as previously described^46^.

### Alizarin red experiment

BMSCs cultured in 10% α-MEM or osteogenic media were stained with Alizarin Red at Day 20 of culture post rAAV9 or rAAV9-NEM transduction. Media was aspirated and cells were washed with 1× PBS. Following 20 min fixation with 4% paraformaldehyde (PFA), 1 mL of Alizarin Red solution (1.0%) was added to cultures for 20 min at room temperature under gentle rocking. 4–5 washes were performed with DI water to remove excess Alizarin Red, and images were taken for analysis via MetaMorph Software^47^.

### Tissue processing, microscopy, and immunofluorescence

For AAV9/9-NEM in vivo studies, liver, spleen, heart, lungs, and hindlimbs were collected from mice and fixed in 4% PFA for 48 h at 4 °C, then transferred to 20% sucrose for 24 h, and then to 25% sucrose at 4 °C. Livers and hindlimbs were embedded in OCT or a 1:1 ratio of OCT and 25% sucrose^11,12^. Tissues were imaged on a Zeiss Axio Observer 7 microscope at 10× and 20× objectives. Fluorescent tissue area was assessed via MetaMorph software at the Indiana Center for Biological Microscopy^46^.

Immunofluorescence was performed on sequential liver and hindlimb sections for mouse Albumin (Proteintech, 16475-1-AP, 1:500 dilution), F4/80 (BioxCell, BE0206, 1:200 dilution), and Endomucin (EMCN, sc-53940, 1:100). A donkey anti-rat or anti- rabbit secondary antibody Alexa-fluor 647 (Invitrogen A78947 & A32795, 1:500) was applied prior to imaging. For Osteocalcin (Ocn, Proteintech #23418-1-AP, 1:150) staining, we performed a similar protocol; a donkey anti-rabbit secondary antibody Alexa 647 (Invitrogen A32795, 1:500) was used before imaging. For immunofluorescence analyses, “% Positive Area” was calculated using color thresholds for red (mScarlet), green (eGFP), yellow (mScarlet & eGFP), or purple (Alexa 647) on MetaMorph software. The entire image (Area = 6.1E^6^ pixels) was analyzed for a specific color area to get the % Positive area. 2–3 images per tissue were analyzed and averaged for the final graphs.

### Flow cytometry

Bone marrow from AAV9/9-NEM studies were collected in the same manner as described in BMSC differentiation section from femurs, tibiae, and humeri. After centrifugation, red blood cells (RBC) were lysed using a 1× RBC Lysis Buffer (Biolegend #420301) for 5 min on ice. The reaction was stopped by adding Cell Staining Buffer (1× PBS with 0.1% BSA and 10 mM HEPES). Tubes were then centrifuged for 5 min at 350×*g*. After discarding the supernatant, 1 mL of fresh Cell Staining Buffer was added to marrow samples. Proper volumes of flow antibodies (Table 2) were added to each sample, and incubation lasted 20 min on ice in the dark. Tubes were then centrifuged for 5 min and 350×*g*, supernatant discarded, and fresh Cell Staining Buffer added to wash samples; this was performed twice^24,25,48,49^.

**Table 2:** Flow cytometry antibody list.

Flow marker	Fluorophore	Volume per 1 mL bone marrow (μls)	Concentration (mg/mL)	Company	Catalogue #
Cd45	BV605	2.50	0.5	Biolegend	103155
Cd31	BV785	1.25	0.25	Biolegend	102435
Cd34	PE/Cy7	2.50	0.5	Biolegend	128617
Cd90	APC-Fire	6.25	1.25	Biolegend	105347
Cd105	Pacific Blue	2.00	1.00	Biolegend	120412
Cxcr4	APC	2.50	0.5	Biolegend	146507

Samples were analyzed using a BD LSRFortessa. For negative control samples and single-color control samples, 30,000–50,000 events were recorded. For experimental samples, 0.5–1 million events were recorded. Forward Scatter Height vs. Forward Scatter Area plot was used to assess singlets, and Forward Scatter Area vs. Side Scatter Area plot was used to further identify cells for protein analyses. Compensation was performed under guidance of the Flow Cytometry Resource Facility (FCRF) at IUSM. Raw data files were analyzed using FlowJo_v10.8.1.

### Statistics

GraphPad Prism 9.1.2 software was used to calculate all statistics. Unless otherwise stated, data are presented as means ± the standard error mean (SEM). For experiments comparing more than 2 groups, one-way ANOVA was used with a Dunnett’s post-hoc test unless stated differently in a specific figure legend. For all tests, a p value less that 0.05 was considered significant.

## Supplementary Information

Supplementary Information.

Supplementary Figures.

## Data Availability

The datasets used and/or analyzed during the current study are available from the corresponding author on reasonable request.
