# Supplementary material for: Chemical modification of AAV9 capsid with N-ethyl maleimide alters vector tissue tropism
Source: Sci Rep. 2023 May 25;13:8436. doi: 10.1038/s41598-023-35547-0 (PMC10212940; doi:10.1038/s41598-023-35547-0)
Supplement: Supplementary file 1 — Supplementary Information. [file 41598_2023_35547_MOESM1_ESM.docx]

**Original Gels and Blots Images of Fig1.**


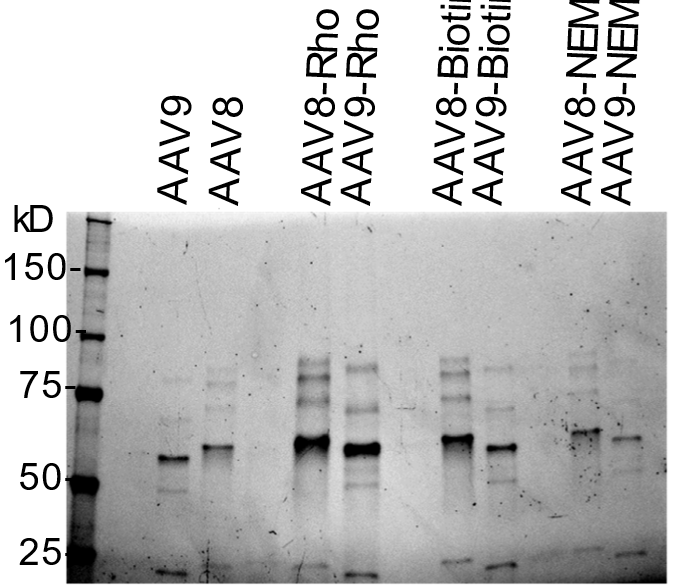
(Shown in Fig1)


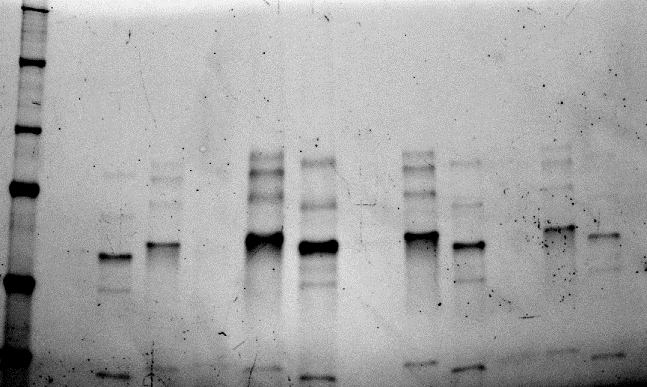

(original image without labels)


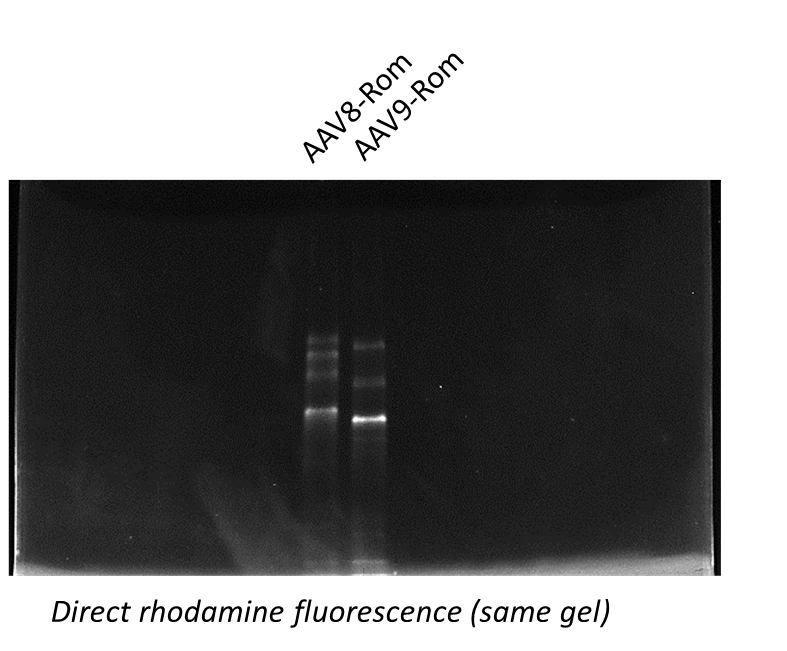


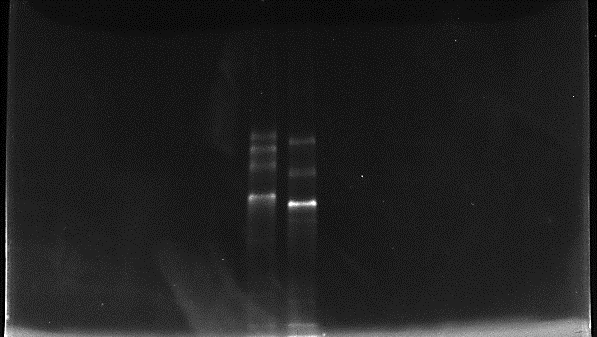
(original image)


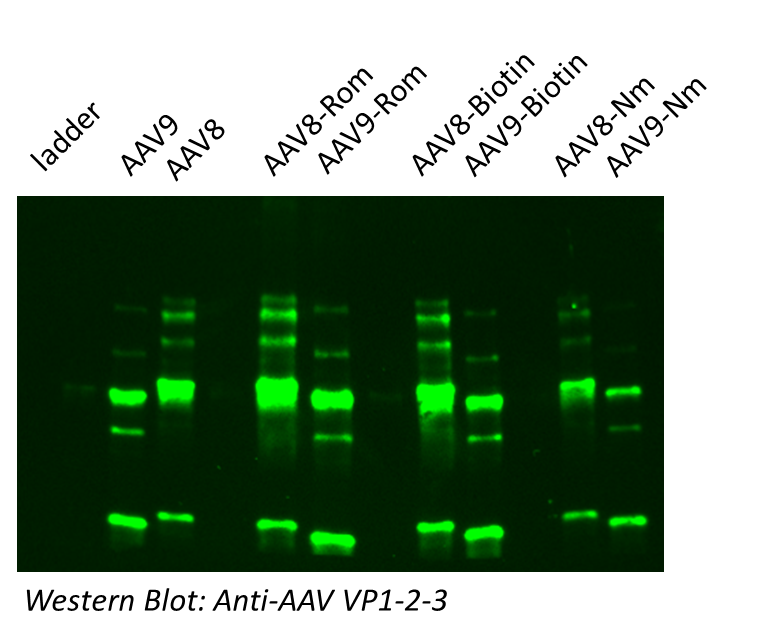


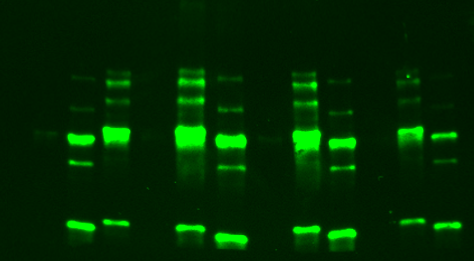


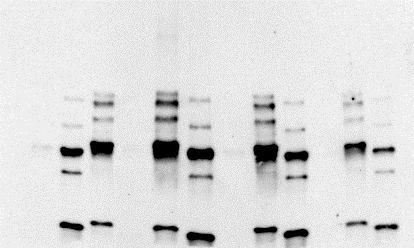

(same blot as above but in gray scale)


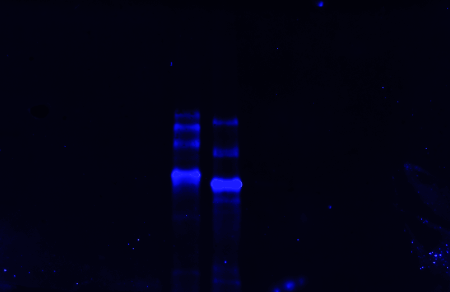


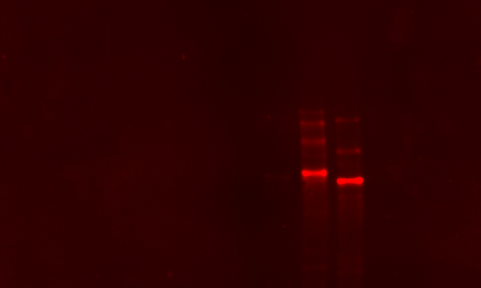


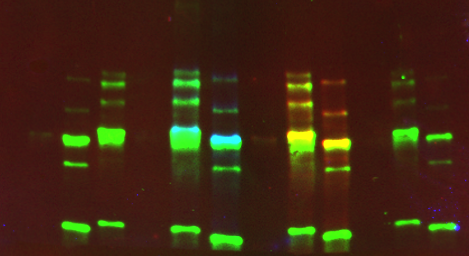


*Western Blot:*

*Blue: Rhodamine fluorescence*

*Red: Anti-Biotin IR-dye-Streptavidin*

*Green: Anti-AAV VP1-2-3*
