# Supplementary material for: Chemical modification of AAV9 capsid with N-ethyl maleimide alters vector tissue tropism
Source: Sci Rep. 2023 May 25;13:8436. doi: 10.1038/s41598-023-35547-0 (PMC10212940; doi:10.1038/s41598-023-35547-0)
Supplement: Supplementary file 2 — Supplementary Figures. [file 41598_2023_35547_MOESM2_ESM.docx]

*Supplemental Information*

**Chemical Modification of AAV9 Capsid with N-ethyl Maleimide Alters Vector Tissue Tropism**

Patrick L. Mulcrone^1†^, Anh K. Lam^1†^, Dylan Frabutt^1^, Junping Zhang^1^, Matthew Chrzanowski^2^, Roland W. Herzog^1^, and Weidong Xiao^1*^

†Authors contributed equally.

^1^ Department of Pediatrics, Herman B Wells Center for Pediatric Research, Indiana University School of Medicine, Indianapolis, IN, 46202, USA

^2^ Lewis Katz School of Medicine, Temple University, Philadelphia, PA 19140, USA

*Corresponding author email: xiaow@iu.edu


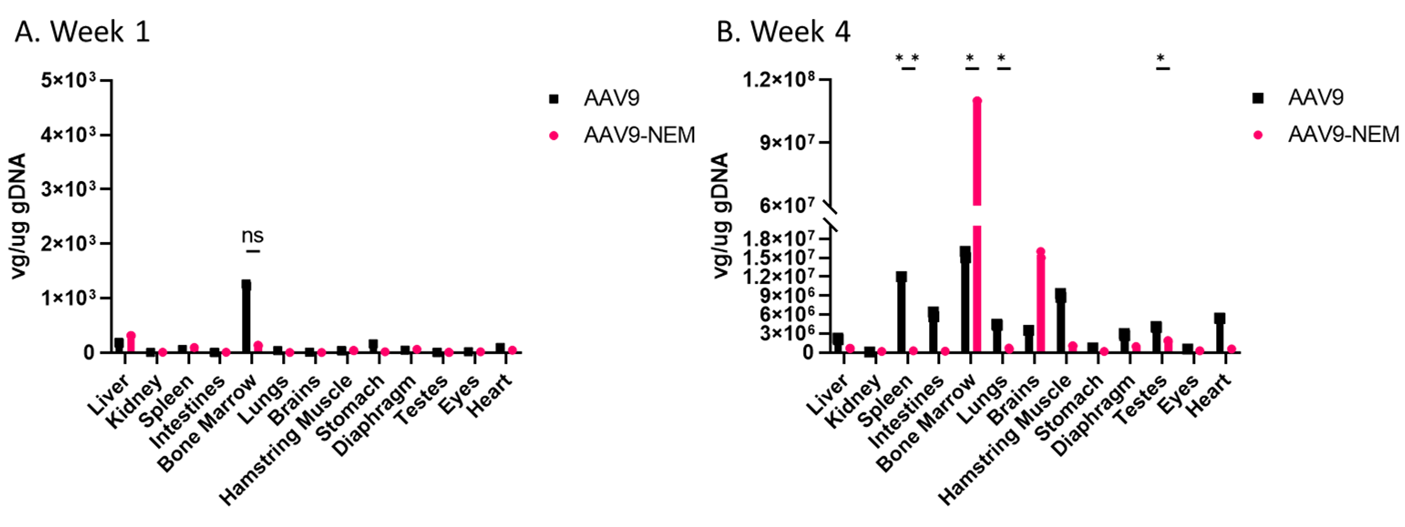


Figure S1. In vivo biodistribution of AAV9 vs AAV9-NEM in WT Balb/c mice at week 1 and week 4 post injection. Multiplexed qPCR designed to target the unique barcode packaged by AAV9 or AAV9-NEM of DNA extracted from different tissues. Normalized data are shown in Figure 3. N = 2 mice for analysis. Comparison via two-way ANOVA. *= p<0.05, **= p<0.01.


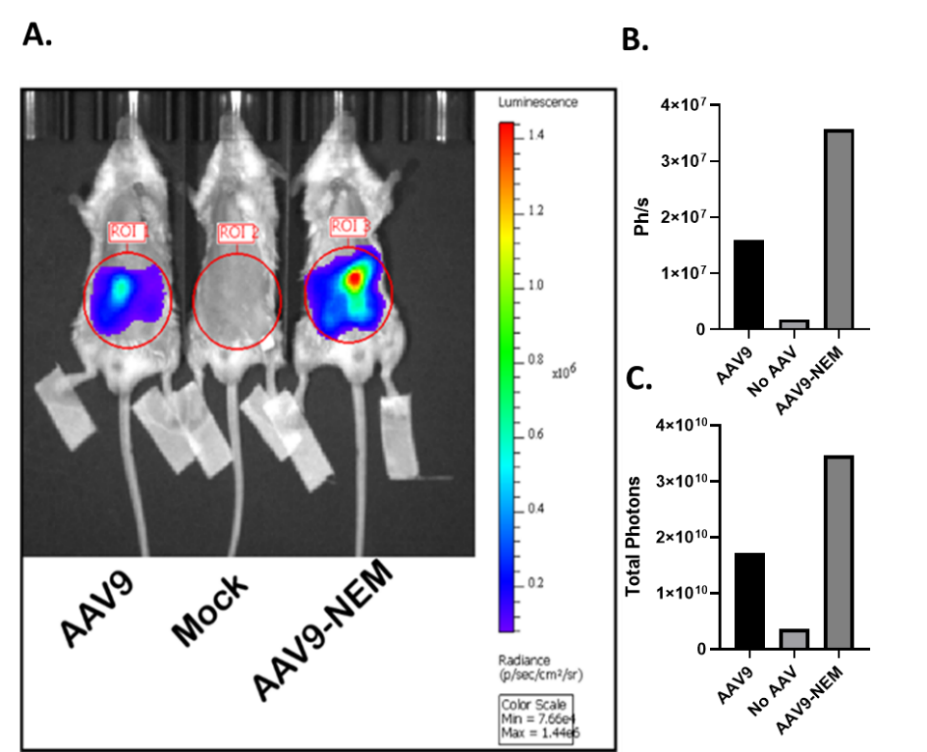


Figure S2: AAV9-NEM-GLuc Emits a Brighter Signal in vivo Compared to AAV9-gLUC. A) Representative image of the mice after substrate injection. B & C) A greater than 2-fold difference measure in Cmax of radiance and Area under the curve (AUC) of the radiance readouts in the AAV9-NEM mouse.


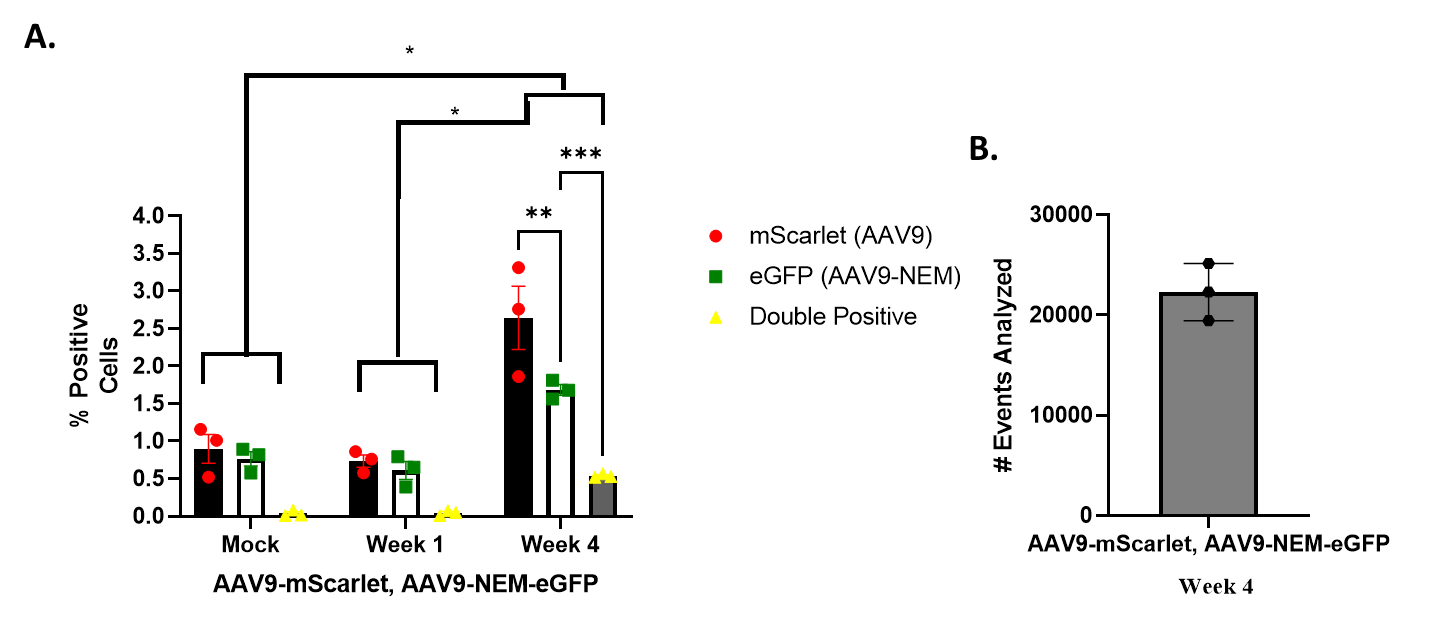


Figure S3: Exchange of Transgenes Does Not Alter AAV9, AAV9-NEM Positivity in Mouse Bone Marrow. A) Mock, Week 1, and Week 4 analyses. Week 4 graphs correlate with Fig. 5A. B) Number of events analyzed for Week 4. N=3 mice, average of 2 bone marrow samples/ mouse. Comparison via two-way ANOVA. *= p<0.05, **= p<0.01, ***= p<0.001


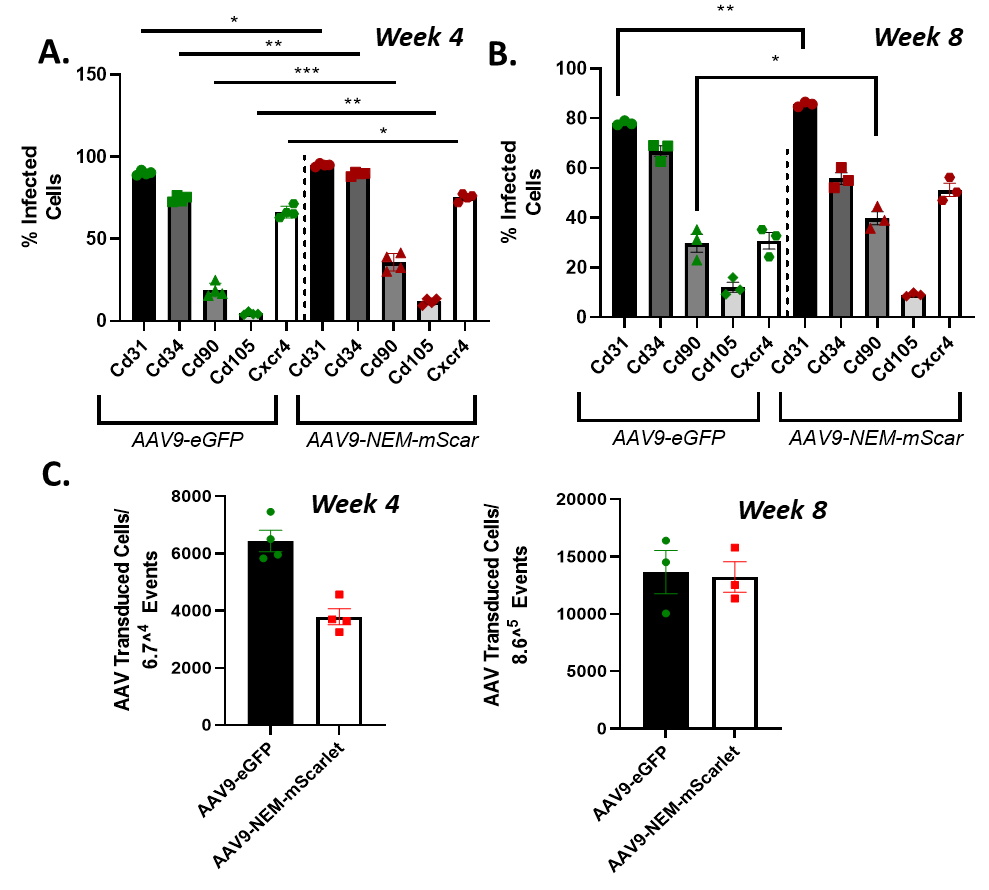


Figure S4: Percentages of AAV9 and AAV9-NEM Transduced Bone Marrow Cells Expressing Vascular and MSC Proteins. A) Week 4 analysis, and B) Week 8 analysis of Cd31, Cd34 Cd90, Cd105, and Cxcr4 single-positive bone marrow. Comparisons are via Paired T-test. C) Absolute number of eGFP or mScarlet-positive bone marrow cells analyzed for Panels A & B, and Figure 5A. N=3-4 mouse bone marrow/ group.


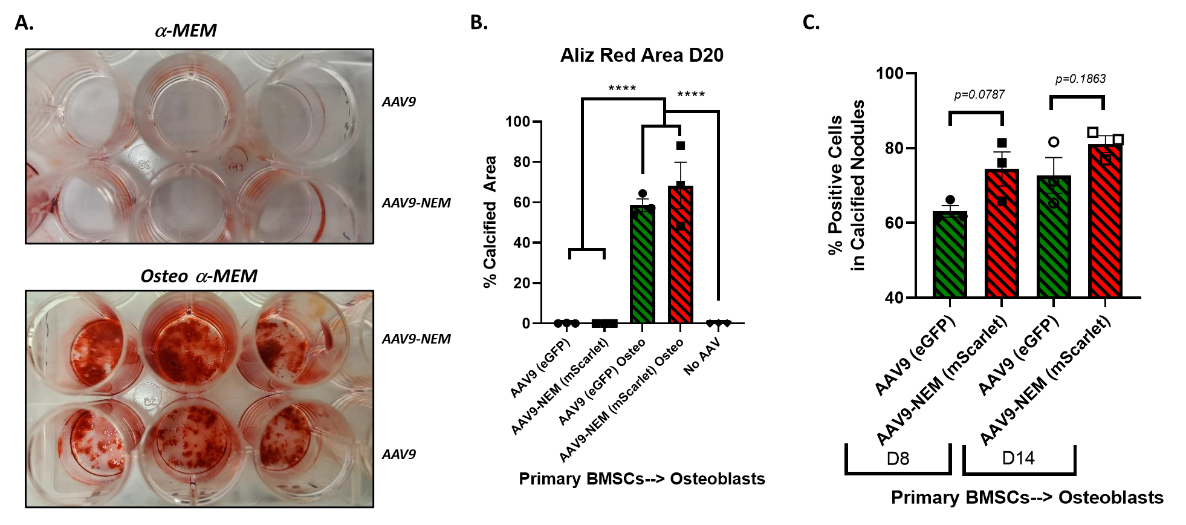


Figure S5: AAV9 and AAV9-NEM-transduced Cells Localize to Calcified Nodules of Mouse Ob-BMSC Cultures. A) Endpoint images of BMSC cultures (Day 20). B) Calcified area analysis based on % red via Alizarin Red staining. One-way ANOVA comparisons. C) Area of GFP or mScarlet positive cells in calcified nodules seen in Figure 4C-F. Slightly higher % observed in AAV9-NEM transduced cultures. T-Test used for comparison, N=3 replicates for each group.

**
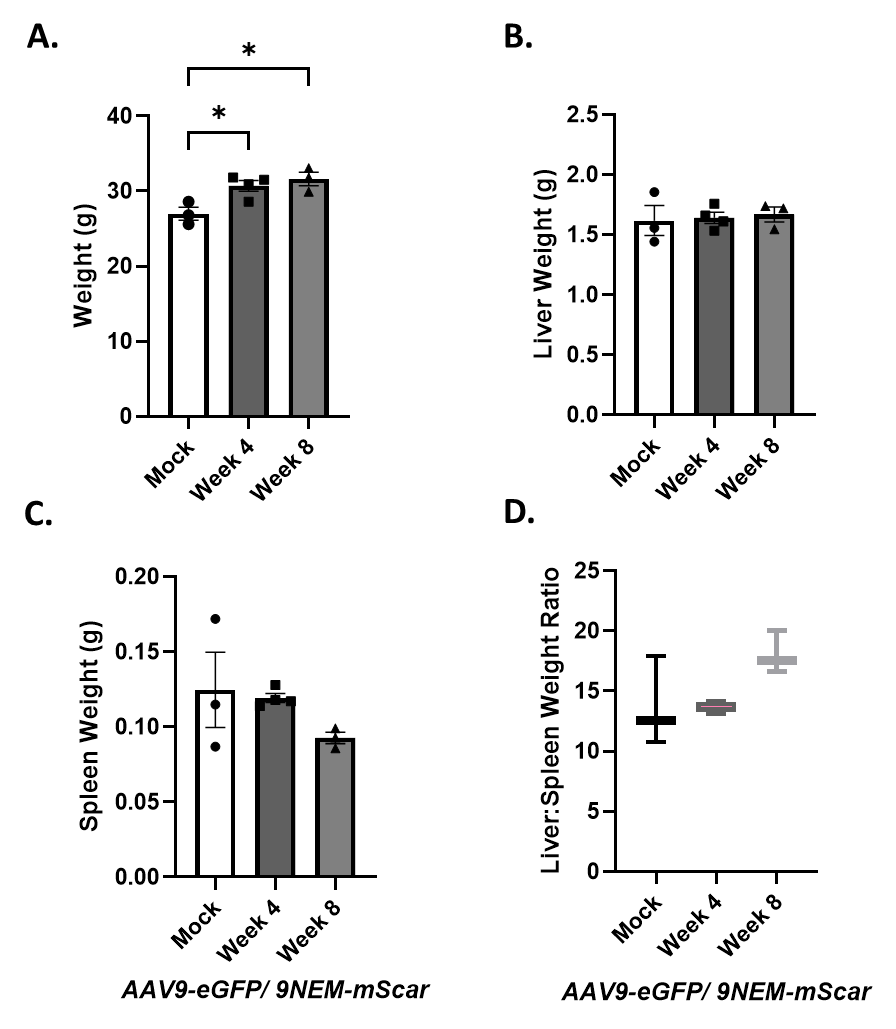
**

Figure S6: Mouse, Liver, and Spleen Weights for AAV9-eGFP/ AAV9-NEM-mScarlet in vivo Study. A) Endpoint whole body weights for mice. One-Way ANOVA, *=p<0.05. B) Liver weights, C) Spleen weights, and D) Liver to Spleen weight ratios. All groups compared by One-way ANOVA. N=3-4 mice/ group. AAV9-eGFP/9NEM-mScar refers to the AAV concoction injected into the mice via tail vein injection. “Mock” animals were injected with sterile 1X PBS.
